# *Psidium guajava* mediated green synthesized cobalt oxide nanoparticles dispersed on reduced graphene oxide for electrocatalytic water splitting†

**DOI:** 10.1039/d5ra00040h

**Published:** 2025-05-01

**Authors:** Sumera Akram, Shabbir Hussain, Muhammad Arif, Mirza Haider Ali, Muhammad Tariq, Abdur Rauf, Khurram Shahzad Munawar, Hamad M. Alkahtani, Amer Alhaj Zen, Syed Adnan Ali Shah

**Affiliations:** a Institute of Chemistry, Khwaja Fareed University of Engineering and Information Technology Rahim Yar Khan 64200 Pakistan shabbir.hussain@kfueit.edu.pk shabchem786@gmail.com; b Institute of Chemical and Environmental Engineering, Khwaja Fareed University of Engineering and Information Technology Rahim Yar Khan 64200 Pakistan; c Department of Chemistry, Lahore Garrison University Lahore Pakistan; d Institute of Chemical Sciences, Bahauddin Zakariya University Multan Pakistan; e Department of Chemistry, University of Sahiwal Sahiwal Pakistan; f Institute of Chemistry, University of Sargodha 40100 Pakistan; g Department of Chemistry, University of Mianwali 42200 Pakistan; h Department of Pharmaceutical Chemistry, College of Pharmacy, King Saud University P. O. Box 2457 Riyadh 11451 Saudi Arabia; i Chemistry & Forensics Department, Nottingham Trent University Clifton Campus Nottingham Ng11 8NS UK; j Faculty of Pharmacy, Universiti Teknologi MARA Cawangan Selangor Kampus Puncak Alam Bandar Puncak Alam Selangor D. E. 42300 Malaysia; k Atta-ur-Rahman Institute for Natural Product Discovery (AuRIns), Universiti Teknologi MARA Cawangan Selangor Kampus Puncak Alam Bandar Puncak Alam Selangor D. E. 42300 Malaysia

## Abstract

In this research, we synthesized (Co_3_O_4_)_aq_ and (Co_3_O_4_)_et_ nanoparticles (NPs) utilizing aqueous and ethanolic extracts, respectively, of *Psidium guajava* leaves. The biosynthesized NPs were sonicated with reduced graphene oxide (rGO) to produce rGO@(CO_3_O_4_)_aq_ and rGO@(Co_3_O_4_)_et_ nanocomposites (NCs) and their respective calcined (700 °C) products rGO@(CO_3_O_4_)_aqc_ and rGO@(Co_3_O_4_)_etc_. The nanomaterials (NMs) were characterized through XRD, FTIR, UV-visible spectroscopy, SEM, TGA, and DSC analyses. They exhibited crystallite sizes of 10–15.4 nm and band gaps of 5.1–5.9 mV. Their surfaces were coated with organic moieties from plant extracts. TGA and DSC analyses showed the endothermic loss of moisture and exothermic evolution of organic contents. SEM images revealed the rough and porous surfaces of NPs, making them efficient catalysts for water splitting. Linear swap voltammetry (LSV) measurements for the oxygen evolution reaction (OER) and hydrogen evolution reaction (HER), Tafel slopes and double layer capacitance (*C*_dl_) values reflected a decrease in electrocatalytic water splitting efficiency in the following order: rGO@(Co_3_O_4_)_aq_ > (Co_3_O_4_)_aq_ > rGO@(Co_3_O_4_)_aqc_ and rGO@(Co_3_O_4_)_et_ > (Co_3_O_4_)_et_ > rGO@(Co_3_O_4_)_etc_. Each aqueous extract-derived nanomaterial was electrocatalytically more active than its respective ethanolic extract-derived counterpart. Moreover, the non-calcined rGO decorated Co_3_O_4_ products showed superior electrocatalytic performance compared with their calcined counterparts and therefore, can be recommended as the best choices for electrocatalytic water splitting applications.

## Abstract 2

*Psidium guajava* mediated rGO@Co_3_O_4_ nanocomposites show exceptional electrocatalytic water splitting potential, as reflected from LSV HER/OER & *C*_dl_ data. Their rough and porous surfaces, make them ideal candidates for sustainable energy applications.

## Graphical abstract

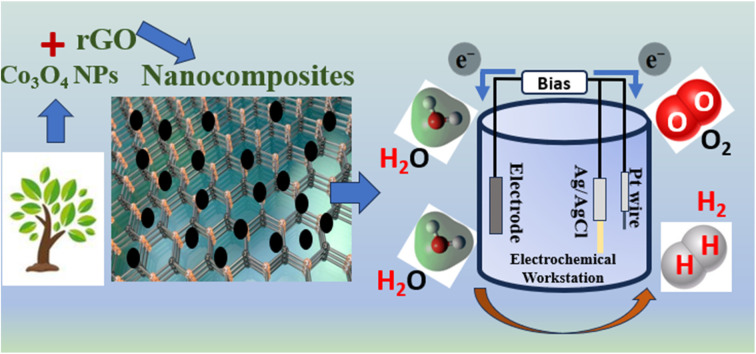

## 1 Introduction

Cobalt oxide nanoparticles (NPs) have attracted the interest of researchers in recent years owing to their non-expensive nature and catalytic capabilities equivalent to those of expensive metals such as Pt and Pd. Therefore, they have extensive applications in catalysis, energy storage devices, and gas sensing devices.^1^ They have emerged as appealing options for electrocatalysis owing to their high catalytic efficiency, stability, and cost-effectiveness.^2^ They often exist in the form of Co_3_O_4_ or CoO and exhibit remarkable catalytic^3^ and electrochemical properties.^4^ They are also important in the renewable energy industry owing to their utilization in energy storage/conversion systems, including supercapacitors and lithium-ion batteries.^5^ Currently, water splitting is an important process for sustainable energy production as it allows for the production of clean and renewable hydrogen fuels. One promising approach to achieve efficient and cost-effective water splitting is the use of electrocatalysts, such as cobalt-oxide NPs. The use of cobalt-oxide as an electrocatalyst for water splitting has been widely investigated considering its high catalytic activity, mechanical strength, stability, easy availability and low cost.^2^

Earlier studies have reported that transition metal oxides display promising properties such as high specific surface areas and diverse morphologies. However, their practical applications are limited by certain drawbacks that can be addressed by integrating carbon-based materials, especially graphene oxide (GO) with metal oxides (MOs). Such synergistically integrated nanocomposites combine the good electrochemical potential and rich redox activity of metal oxides with the ultra-thin structure, large specific surface area and exceptional thermal/electrical conductivity of GO. GO–MO hybrid materials exhibit superior specific capacitance and cyclic stability in ultracapacitors compared with their individual components and offer excellent potential for advancing next-generation supercapacitor materials.^6^ They exhibit large surface areas, high chemical stability, excellent thermal and electrical conductivity, and have attracted significant attention in energy conversion processes like oxygen reduction reactions (ORR) and hydrogen evolution reactions (HER) and in energy storage devices like supercapacitors, batteries,^7^ solar cells and fuel cells.^8^ Among the carbon-based materials, graphene and carbon nanotubes (CNT) are regarded as the new-generation and state-of-the-art nano-reinforcement for metals owing to their outstanding multifunctional features, extraordinary mechanical properties, and unique nanostructures.^9^ However, graphene has attracted more attention than CNTs owing to its higher aspect ratios, 2D flat geometry, unique surface texture (capability to interlock mechanically with the matrix), cost-effective production,^10^ better surface properties (under ambient conditions) and higher selectivity against interferences.^11^ Currently, GO has emerged as a versatile substance with its outstanding properties in energy conversion and storage technologies.^8^ GO–MO hybrids display improved surface area compared with their individual constituents, resulting in better charge separation properties, high and selective adsorption capacity towards metal ions and organic species, and photocatalytic degradation of pollutant dyes and pathogens. They are also effective in energy storage, water purification, antibacterial applications, controlled drug release and selective destruction of cancerous cells.^12^ They exhibit improved conductivity, stability, and reactivity by leveraging the strengths of GO and metal oxides, making them promising candidates for advanced materials in numerous technological domains, including electronic devices, catalysts, sensors and energy storage devices. GO contributes to the improved surface functionality, mechanical strength and excellent conductivity, whereas metal oxides are attributed to specific chemical and electronic properties.^8^ GO finds significance due to its increased polarity and compatibility with other nanomaterials, making it an important component in composite electrodes.^6^ Reduced graphene oxide (rGO) provides a flat and broad surface with several functional groups and defects, which increases its dispersion and integration with metal oxides. The increased roughness factor and surface area significantly promotes its electrochemical potential and electrocatalytic efficiency.^13^ rGO-metal oxide nanocomposites combine the pseudocapacitive properties of metal oxides with the conductivity of rGO^14^ and are highly suitable candidates for supercapacitors due to their excellent mechanical behavior, good chemical stability, superior electrical conductivity and high surface area.^15^ They find multifaceted applications in designing and fabricating smart materials due to their unique photochemical-, photocatalytic-, sensing-, mechanical-, electrical-, optical- and energy-storing capacities.^16^

Cobalt oxide-rGO nanocomposites were earlier reported as electrocatalysts for OER,^17^ electrode materials for supercapacitors,^18^ anode materials for lithium ion batteries,^19^ advanced multifunctional microwave absorbers,^20^ acetone sensors^21^ and sensitive electrochemical detectors of trace Pb(ii) ions in environmental samples.^22^ Cobalt NPs have a high surface area to volume ratio and display excellent catalytic activity,^23^ while rGO exhibits good carrier transportation, excellent thermal and chemical stability, electrical conductivity, hydrophobicity, and safety, high mechanical strength, substantial specific surface area,^24^ and good durability and performance, for advanced materials.^25^ Under moderate hydrothermal conditions, the surface contact between graphene oxide (GO) sheets and Co^2+^ ions may be altered by deoxygenating a few layers of GO.^26^ The presence of oxygen-containing functional groups on rGO surface facilitates its better bonding with Co_3_O_4_ NPs. These groups act as anchoring sites, helping in the uniform distribution of Co_3_O_4_ NPs and improving the composite's stability and performance. On the other hand, CNTs have fewer surface functional groups, making it harder for them to interact with metal oxides in a similar way. The choice of rGO over GO for synthesizing composites stems from its superior electrical conductivity, higher mechanical stability, and improved chemical resistance, which are crucial for high-performance applications.^27^ Due to the greater portion of sp^3^ hybridized carbons linked with the oxygen-containing moieties, GO is typically insulating and displays a very high sheet resistance. The sheet resistance of GO is greatly lowered after its reduction into rGO, hence converting it into a semiconductor or even into a graphene-like semimetal.^12^

There are numerous physical and chemical processes for creating NPs, but green synthetic pathways are environmentally friendly and the most suitable methods^28^ because they reduce the use of harmful chemicals, solvents, and other synthetic agents and minimize the toxic environmental effects associated with chemical synthesis.^29^ Green synthesis has received substantial attention in recent years, especially in the field of materials science^30^ and researchers are constantly exploring new and innovative nano-synthetic methods *via* the use of renewable energy sources such as plant extracts.^31^ Plants are rich sources of antioxidants including phenolics, flavonoids, fatty acids, *etc.*;^32^ therefore, they can be employed as reducing agents for the conversation of metal salts into their respective NPs.

In the present study, we have employed the aqueous and ethanolic extracts of *Psidium guajava* leaves to synthesize cobalt oxide (Co_3_O_4_) NPs and then their nanocomposites with rGO. The investigated biosynthesis can be attributed to the diverse phytochemical profile of *P. guajava* which includes flavonoids, polyphenols, terpenoids, and tannins.^33^ In particular, flavonoids and polyphenols are known for their strong reducing and capping abilities, allowing for the controlled growth and stabilization^34^ of cobalt ions during the production of NPs. The biosynthesized nanomaterials were analyzed by XRD studies, FTIR analysis, UV-visible spectroscopy, SEM studies, TGA and DSC analyses. They were also tested for their electrocatalytic water splitting potential by linear sweep voltammetry (LSV), HER and OER measurements and their surface areas were compared in terms of double-layer capacitance (*C*_dl_). This research aimed to comprehensively explore the diverse and ever-expanding utilization of cobalt oxide nanoparticles for water splitting, highlighting their immense potential for driving innovation and addressing contemporary challenges. The main objective was to develop an electrode with enhanced electrochemical performance through combination of cobalt oxide with graphene material.

## 2 Experimental

### 2.1 Materials and methods

Cobalt nitrate hexahydrate and ethanol were purchased from BDH Laboratory Supplies Poole, England. Nickel foam, *N*-methyl-2-pyrrolidone (NMP), potassium hydroxide (KOH), and polyvinylidene fluoride (PVDF) were procured from Sigma-Aldrich, USA and used for electrochemical studies. A reported procedure was used to produce reduced graphene oxide (rGO).^35^ A biosynthesized (Co_3_O_4_)_aq_ or (Co_3_O_4_)_et_ sample was put in a Gooch crucible and calcined for 2 h at 400 °C in a muffle furnace whereas rGO@(CO_3_O_4_)_aq_ and rGO@(Co_3_O_4_)_et_ were calcinated at 700 °C to leave behind rGO@(CO_3_O_4_)_aqc_ and rGO@(Co_3_O_4_)_etc_, respectively.

Powder XRD analysis was performed using a PANalytical X'Pert Pro X-ray diffractometer. Other analytical instruments include Shimadzu FTIR-8400 Fourier transform infrared spectrometer, CE 7200 double-beam UV-visible spectrophotometer for UV-visible spectroscopy and an Emcrafts Cube 1100 for SEM analysis. Thermogravimetric analysis (TGA) and differential scanning calorimetry (DSC) were performed from ambient temperature to 1000 °C using a TA Instruments Discovery 650 SDT simultaneous thermal analyzer, with a heating rate of 10 °C per minute under a 99.999% nitrogen atmosphere and an average flow rate of 50 mL min^−1^ was maintained. An electrochemical workstation (potentiostat, CHI 760 E, CH Instrument Co., USA) was used to conduct the electrochemical studies.

The electrochemical experiments were conducted using a standard three-electrode system, with nickel foam as the working electrode, a platinum wire as the counter electrode and an Ag/AgCl electrode filled with saturated KCl as a reference electrode which was calibrated to the reversible hydrogen electrode (RHE) in a 1 M KOH-saturated electrolyte. To activate the catalyst-coated electrodes, 20 cycles were run by performing cyclic voltammetry (CV) at a 0.1 V s^−1^ scan rate within the 0.2–0.8 V *vs.* Ag/AgCl potential range. Additional CVs were recorded at various scan rates (100, 80, 60 and 40 mV s^−1^) to determine the double-layer capacitance (*C*_dl_).

### 2.2 Methodology

#### 2.2.1 Collection and identification of plant

**Fig. 1:**
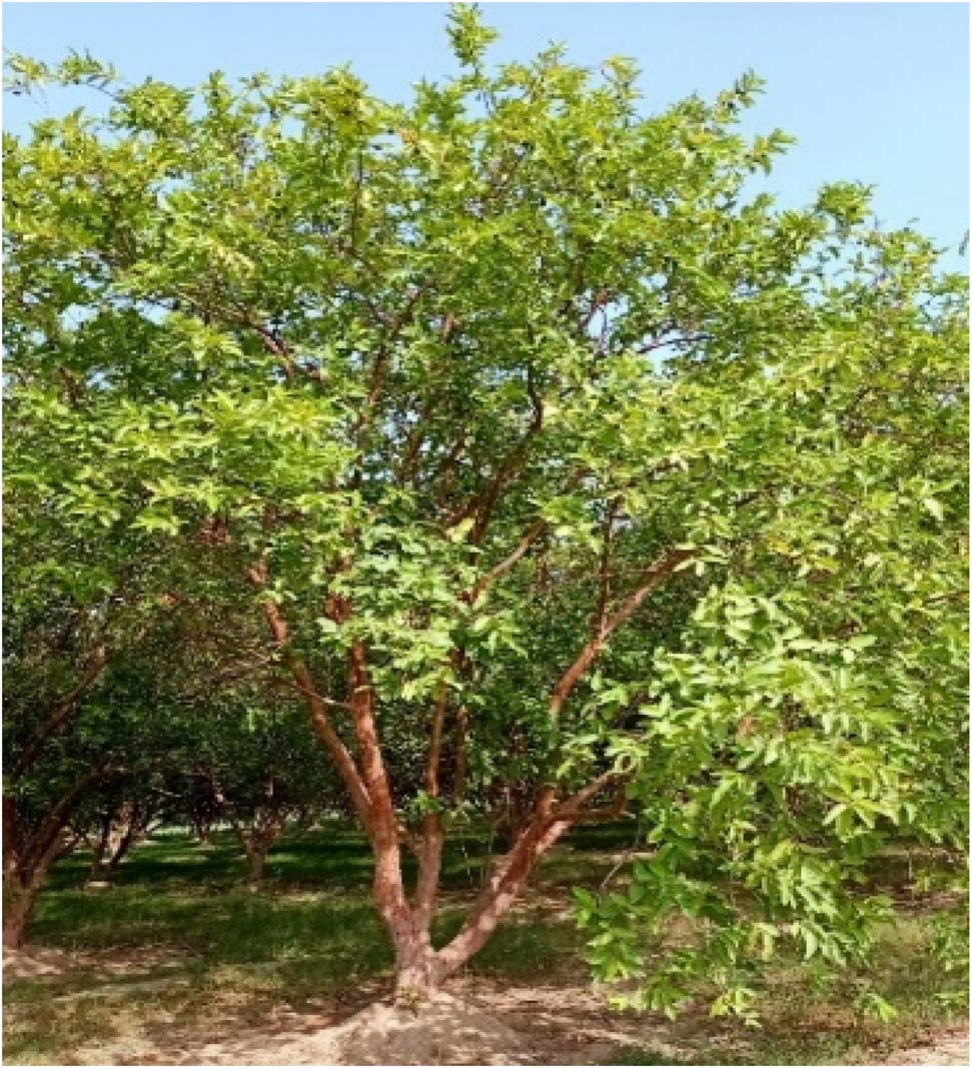
*Psidium guajava* (guava) plant.

The leaves of *Psidium guajava* plant (Fig. 1) were collected from a local guava garden, near Chak No. 84/P, Rahim Yar Khan (Punjab, Pakistan) on August 6, 2022. The plant species was identified by the Department of Life Sciences, Khwaja Fareed University of Engineering and Information Technology, Rahim Yar Khan 64200, Pakistan. The collected leaves of *P. guajava* were washed thrice with water to rinse off the dirt and impurities. Afterward, they were dried under shade for 7 days and ground into a fine powder, which was then stored in a dry, clean and air-tight jar for further usage.

#### 2.2.2 Preparation of aqueous/ethanolic extracts of *Psidium guajava* leaves

A 5 g powder of *P. guajava* leaves was added to 250 milliliters of distilled water in a beaker. Then, the beaker was covered with aluminum foil, and its contents were vigorously stirred at 70 °C for 3 h until the solution changed color from clear to dark brown. After cooling, the mixture was filtered using Whatman filter paper (grade 1). The freshly prepared aqueous extract (filtrate) was subsequently used for the synthesis of (Co_3_O_4_)_aq_ nanoparticles.

Five g of *P. guajava* leaf powder was mixed with 250 mL of ethanol, stirred at 40 °C for 30 min, followed by filtration to obtain the ethanolic extract (filtrate), which was employed for the synthesis of (Co_3_O_4_)_et_ NPs.

#### 2.2.3 Green synthesis of (Co_3_O_4_)_aq_ and (Co_3_O_4_)_et_ nanoparticles

One g of cobalt nitrate hexahydrate [Co(NO_3_)_2_·6H_2_O] was homogenously mixed with 150 mL of distilled water in a beaker and then 100 mL aqueous extract of *P. guajava* leaves was added. Subsequently, the beaker was covered by aluminum foil and its contents were vigorously stirred at 40 °C for 30 min. The mixture was kept overnight to allow the precipitated nanoparticles (NPs) to settle at the bottom of the beaker. The top layer of clear solution was discarded and the residual bottom mixture containing precipitates of NPs was centrifuged at 4000 rpm for 3 min. The obtained precipitates were rinsed with water and then with ethanol before being dried in an oven at 60 °C. Finally, they were calcined for 2 h at 400 °C to produce the final form of (Co_3_O_4_)_aq_ NPs.

**Fig. 2:**
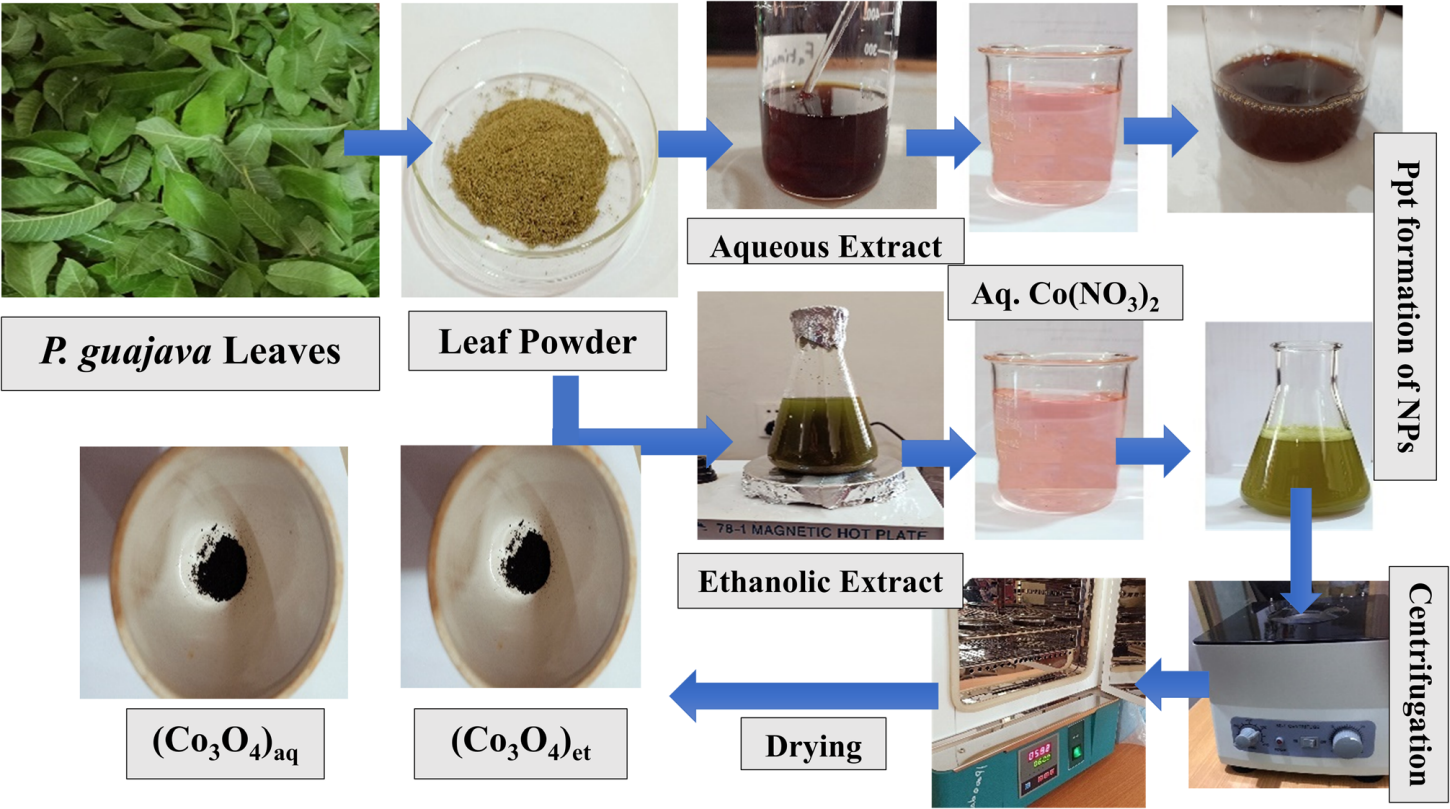
Schematic showing the biogenic synthesis of (Co_3_O_4_)_aq_ and (Co_3_O_4_)_et_ NPs.

The same procedure was followed for the green synthesis of (Co_3_O_4_)_et_ NPs except that the ethanolic extract of *P. guajava* leaves was used instead of the aqueous extract. Fig. 2 summarizes the whole biosynthetic route of (Co_3_O_4_)_aq_ and (Co_3_O_4_)_et_ NPs.

#### 2.2.4 Synthesis of rGO-based Co_3_O_4_ nanocomposites

The rGO-decorated cobalt oxide nanocomposites *i.e.*, rGO@(Co_3_O_4_)_aq_ and rGO@(Co_3_O_4_)_et_, were prepared by sonicating 10% rGO with (Co_3_O_4_)_aq_ and (Co_3_O_4_)_et_ NPs, respectively.

0.01 g of rGO suspension in 50 mL of distilled water was sonicated for 150 min. Then, 0.1 g of (Co_3_O_4_)_aq_ NPs were added and the mixture was subjected to another 3.5 h sonication until a homogeneous mixture was obtained. The as formed nanocomposite was separated by centrifugation (10 000 rpm) for 20 minutes and washed thrice with distilled water, followed by centrifugation each time and dried at 60 °C in an incubator/oven to produce solid rGO@(Co_3_O_4_)_aq_. Finally, rGO@(Co_3_O_4_)_aq_ was calcined at 700 °C for 120 min in a muffle furnace to produce rGO@(Co_3_O_4_)_aqc_.

**Fig. 3:**
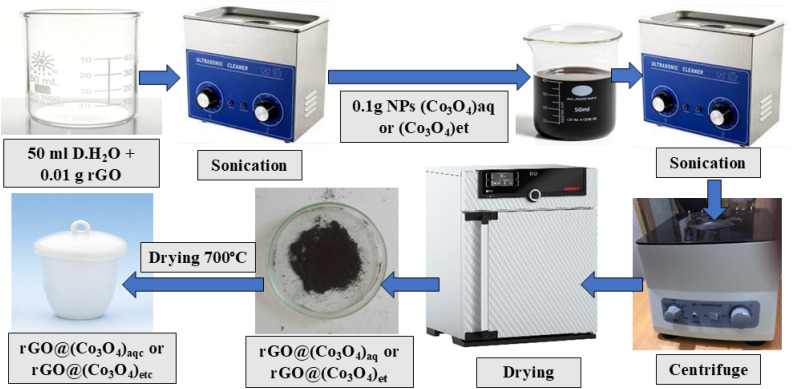
Schematic showing the synthesis of rGO decorated Co_3_O_4_ nanocomposites.

The same methodology (discussed above) was followed for the biosynthesis of rGO@(Co_3_O_4_)_et_ nanocomposites from (Co_3_O_4_)_et_ and rGO. The resulting rGO@(Co_3_O_4_)_et_ product was calcined at 700 °C for 120 min to produce rGO@(Co_3_O_4_)_etc_. Fig. 3 displays the synthetic route for the synthesis of rGO decorated Co_3_O_4_ nanocomposites.

### 2.3 Electrochemical studies

#### 2.3.1 Electrochemical measurements

The OER, HER and CV experiments were performed using a potentiostat (CHI 760 E, CH Instrument Co. USA). Each electrochemical experiment involved a standard three-electrode configuration with an Ag/AgCl electrode (as reference electrode), nickel foam (as working electrode) and a Pt wire (as counter electrode) in a 1 M saturated solution of potassium hydroxide (as the electrolyte). For OER and HER, the applied potential was transformed into overpotential (*η*) from *E vs.* Ag/AgCl using the equation *η* = *E vs.* RHE – 1.23 V = *E vs.* Ag/AgCl – 0.221 V (reversible hydrogen electrode). Linear sweep voltammetry (LSV) curves for OER and HER were obtained at a 10 mV s^−1^ scan rate. These LSV curves were subsequently utilized to derive Tafel slope curves. To activate the catalyst-coated electrodes in the electrolyte, CVs were conducted at 0.1 V s^−1^ scan rate within a 1.2–1.3 V *vs.* RHE potential range. Additional CVs were recorded at varying scan speeds (40, 60, 80, and 100 mV s^−1^). The CVs at different scan rates were used to determine *C*_dl_ values. The double layer capacitance (*C*_dl_) was calculated by plotting Δ*J* = (*J*_a_ − *J*_c_) against numerous scan rates, with the linear slope value being twice *C*_dl_.^36^

#### 2.3.2 Electrode preparation

To activate nickel foam, it is diced into homogeneous strips measuring 2 cm in length and 1 cm in width. The strips are sonicated for an hour after being added to a beaker containing a few drops of HCl and 50 mL of water. They are then dried in an oven at 40 °C for 12 h.

To make the binder, 0.2 g of PVDF was added to 5 mL of *N*-methyl-2-pyrrolidone (NMP), and the mixture was agitated for 24 h.

To prepare the electrode, a China dish containing 0.08 g of a sample's material was utilized. Then one drop of the binder was combined with 0.01 grams of activated carbon and gently blended. The resulting mixture was subsequently applied twice to both sides of the activated nickel foam strips using a small painting brush. Finally, the coated strips were oven-dried for 12 h at 40 °C.^37^

## 3 Results and discussion

### 3.1 Role of *Psidium guajava* leaves in green synthesis of NPs

Compared with conventional techniques, plant-mediated synthesis is a very straightforward and easy approach to produce NPs on a large scale.^29^ In the present study, we employed the leaves extracts of *P. guajava* as sustainable biomaterials for the biosynthesis of cobalt oxide NPs and their rGO decorated NCs. The leaves of this plant are commonly available in the fresh form as well as waste materials. Moreover, their use in the nano-synthesis do not add any toxic materials into the environment. We have employed water and ethanol for the extraction of phytochemicals because both of these solvents are easily available, cost-effective and eco-friendly. They are also acceptable for consumption by human beings and do not add any hazardous residues into the final nano-products, thus maintaining their quality and environmental sustainability. However, the phytochemical's extraction efficiency in these solvents may vary to some extent due to their different nature (water-inorganic and ethanol-organic) and water is somewhat more polar compared with ethanol. Water and ethanol are highly efficient in the extraction of polar compounds, especially phenolics and flavonoids.^29^ The conversion of the ions of a metallic salt into its nanoparticles is believed to occur through the action of polar secondary metabolites, including tannins, glycosides, polyphenolics, triterpenes, terpenes, and flavonoids, which are abundant in *P. guajava* leaves.^34^ The biomolecules present in *P. guava* leaves have functional groups that can coordinate with metal ions and facilitate the formation and stability of NPs.^38^

### 3.2 X-ray diffraction studies

**Fig. 4:**
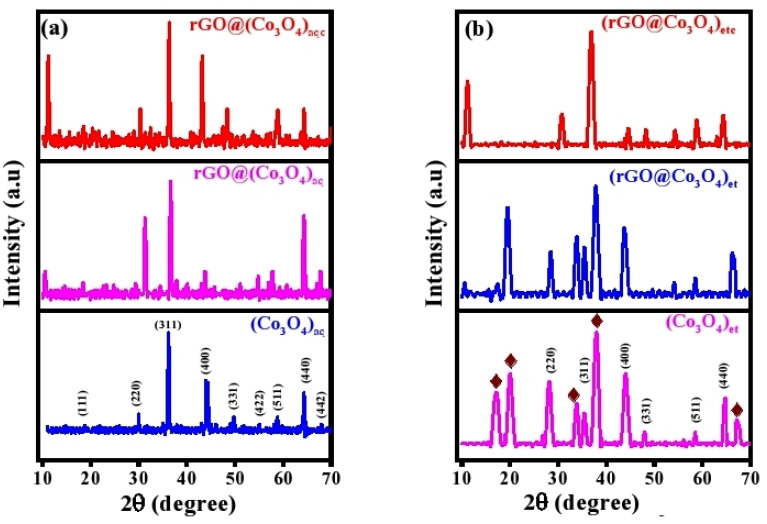
XRD patterns: (a) (Co_3_O_4_)_aq_, rGO@(Co_3_O_4_)_aq_ and rGO@(Co_3_O_4_)_aqc_; (b) (Co_3_O4)_et_, rGO@(Co_3_O_4_)_et_ and rGO@(Co_3_O_4_)_etc_. The peaks represented by the 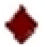 sign indicate impurities *e.g.*, cobalt hydroxide and metallic cobalt.

The synthesized Co_3_O_4_ NPs and their nanocomposites with rGO were subjected to XRD analysis to study their crystallite structures, phase compositions, and crystallite properties. The XRD patterns for (Co_3_O_4_)_aq_, rGO@(Co_3_O_4_)_aq_, and rGO@(Co_3_O_4_)_aqc_ are shown in Fig. 4a, whereas those for (Co_3_O_4_)_et_, rGO@(Co_3_O_4_)_et_, and rGO@(Co_3_O_4_)_etc_ are displayed in Fig. 4b. (Co_3_O_4_)_aq_ exhibited diffraction peaks at 2*θ* values of, 18.72°, 30.03°, 36.26°, 44.17°, 49.78°, 54.90°, 58.72°, 64.41°, and 67.96°, whereas (Co_3_O_4_)_et_ displayed diffraction peaks at 17.07°, 19.88°, 28.16°, 33.75°, 35.36°, 38.20°, 44.01°, 48.01°, 58.45°, 64.69° and 67.29°; these diffraction peaks correspond to the crystallite cubic phase of Co_3_O_4_ and the space group of *Fd*3*m*, which align with JCPDS card no. 01-080-1540. A few extra peaks are also present in (Co_3_O_4_)_et_ sample at 2*θ* values of 17.07°, 33.75°, 38.20°, and 67.29° (represented by the 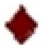 sign in Fig. 4), which are due to the formation of cobalt hydroxide (JCPDS card no. 00-002-0214). The reference XRD spectra of Co_3_O_4_ and cobalt hydroxide are shown in Fig. S1 and S2, respectively of the ESI.†

The 2*θ* values of 10.53°, 18.42°, 31.35°, 36.64°, 38.01°, 43.87°, 54.87°, 58.01°, 64.31° and 67.62° in rGO@(Co_3_O_4_)_aq_ and of 10.66°, 17.29°, 19.50°, 28.53°, 34.01°, 35.37°, 37.78°, 43.98°, 58.01°, 54.23°, 58.45° and 66.51° in rGO@(Co_3_O_4_)_et_ agree with the incorporation of rGO into Co_3_O_4_ in these two nanostructures. The rGO peak is displayed at 10.53° and 10.66° in rGO@(Co_3_O_4_)_aq_ and rGO@(Co_3_O_4_)_et_, respectively. A peak at 43.87° and 43.98° in rGO@(Co_3_O_4_)_aq_ and rGO@(Co_3_O_4_)_et_, respectively can been assigned to metallic cobalt. The diffraction peaks appeared at 2*θ* values of 11.20°, 18.43°, 30.38°, 36.32°, 43.21°, 48.41°, 53.91°, 58.91° and 64.31° in rGO@(Co_3_O_4_)_aqc_ and of 11.26°, 18.29°, 30.74°, 36.97°, 44.60°, 48.22°, 54.44°, 58.86° and 64.49° in rGO@(Co_3_O_4_)_etc_. Both of these calcined samples *i.e.*, rGO@(Co_3_O_4_)_aqc_ and rGO@(Co_3_O_4_)_etc_ represent the purer Co_3_O_4_ phase compared with their non-calcined counterparts *i.e.*, rGO@(Co_3_O_4_)_aq_ and rGO@(Co_3_O_4_)_et_, respectively. A peak at 11.20° in rGO@(Co_3_O_4_)_aqc_ and at 11.66° in rGO@(Co_3_O_4_)_etc_ represents the incorporation of reduced graphene oxide in these two nanomaterials.

The Debye–Scherrer formula, *D* = *Kλ*/*β* cos(*θ*), was used to calculate average crystallite sizes of the nanomaterials. In this equation, *K* represents the shape factor, *λ* is the wavelength of X-ray radiations, *β* denotes the full width at half maximum (FWHM) of the diffraction peak, *θ* is the Bragg angle, and *D* indicates the calculated crystallite sizes. The aqueous extract-derived (Co_3_O_4_)_aq_ possessed the smaller crystallite size (10 nm) compared with its ethanolic extract-derived counterpart (15.4 nm). Actually, the solvent used for extracting bioactive compounds from *P. guajava* leaves (like water *vs.* ethanol) can affect the types and quantities of bioactive compounds present. The results of our study demonstrate that the aqueous extract of *P. guajava* leaves influences the nucleation and growth dynamics of NPs in different ways compared with the ethanolic extract to produce different sized NPs in both cases.^39^ rGO@(Co_3_O_4_)_aq_ and rGO@(Co_3_O_4_)_et_ have shown an average crystallite size of 12 and 11.57 nm, respectively which was further lowered to 11.5 nm in both the calcined nanocomposites *i.e.*, rGO@(Co_3_O_4_)_aqc_ and rGO@(Co_3_O_4_)_etc_. The results of our study clearly demonstrate that the average crystallite sizes of biosynthesized nanomaterials vary depending upon the nature of plant extraction solvent, the doping of Co_3_O_4_ with rGO and the calcination temperature.

### 3.3 FT-IR analysis

**Fig. 5:**
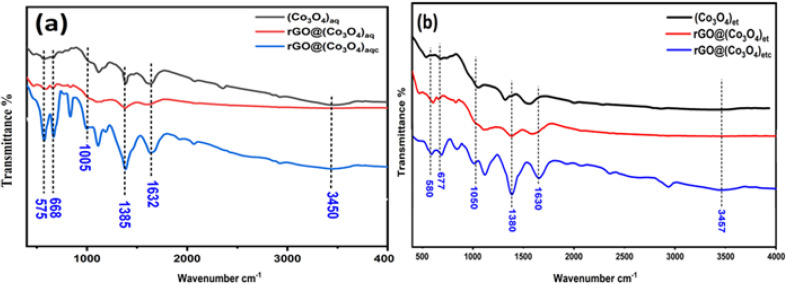
FT-IR spectra: (a) (Co_3_O_4_)_aq_, rGO@(Co_3_O_4_)_aq_ and rGO@(Co_3_O_4_)_aqc_; (b) (Co_3_O4)_et_, rGO@(Co_3_O_4_)_et_ and rGO@(Co_3_O_4_)_etc_.

FTIR analysis was performed in the range of 400–4000 cm^−1^ using the FTIR-8400 spectrometer and the obtained spectra are shown in Fig. 5a and b. The existence of two peaks at 668 and 575 cm^−1^ in each of the three aqueous extract-derived nanomaterials *i.e.*, (Co_3_O_4_)_aq_, rGO@(Co_3_O_4_)_aq_ and rGO@(Co_3_O_4_)_aqc_ (Fig. 5a) and at 677 and 580 cm^−1^ in each of the three ethanolic extract-derived nanomaterials *i.e.*, (Co_3_O_4_)_et_, rGO@(Co_3_O_4_)_et_ and rGO@(Co_3_O_4_)_etc_ (Fig. 5b) correspond to cobalt oxide (Co_3_O_4_) vibrations.^40^ The broader peaks at 3450 and 3457 cm^−1^ are consistent with the presence of hydroxyl groups of phenolic compounds as reported earlier in similar studies on green synthesis using *P. guajava*.^41^ The other major peaks at 1632 cm^−1^ (Fig. 5a) and 1630 cm^−1^ (Fig. 5b) can be assigned to the C=C skeletal, 1385 cm^−1^ (Fig. 5a) and 1380 cm^−1^ (Fig. 5b) to O–H bending, 1005 cm^−1^ (Fig. 5a) and 1050 cm^−1^ (Fig. 5b) to C–O stretching vibration (alkoxy group). These peaks are evidence for the presence of rGO and also confirms the formation of rGO doped cobalt oxide nanocomposites.^42,43^

### 3.4 UV-visible spectrophotometer analysis

**Fig. 6:**
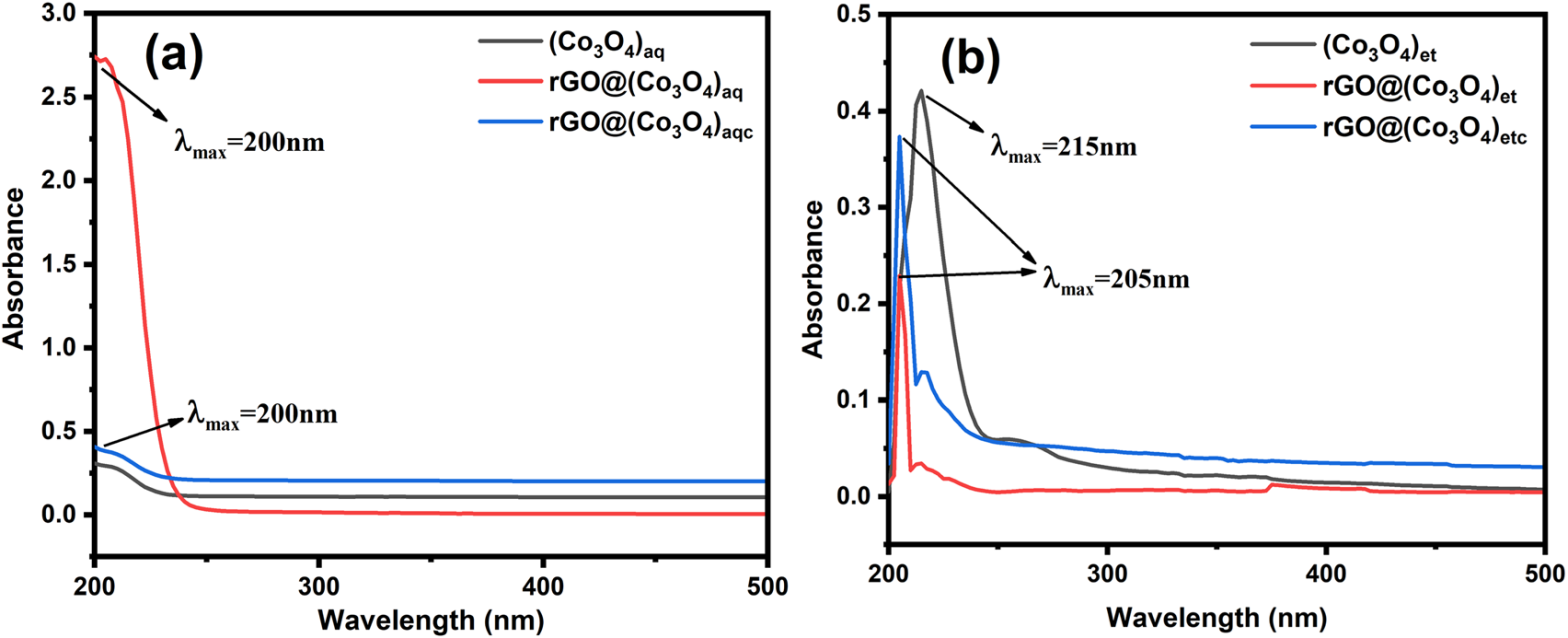
UV-visible spectra: (a) (Co_3_O_4_)_aq_, rGO@(Co_3_O_4_)_aq_ and rGO@(Co_3_O_4_)_aqc_; (b) (Co_3_O4)_et_, rGO@(Co_3_O_4_)_et_ and rGO@(Co_3_O_4_)_etc_.

UV-visible spectra of the investigated nanomaterials were recorded in the range of 200 to 800 nm and are shown in Fig. 6a and b. Co_3_O_4_ NPs and their rGO decorated nanocomposites exhibited a pronounced absorption at 200–275 nm in the visible region. All the aqueous extract-derived nanomaterials *i.e.*, (Co_3_O_4_)_aq_, rGO@(Co_3_O_4_)_aq_ and rGO@(Co_3_O_4_)_aqc_ have a single absorption maximum (*λ*_max_) at 200 nm. (Co_3_O_4_)_et_ showed *λ*_max_ at 215 nm, whereas rGO@(Co_3_O_4_)_et_ and rGO@(Co_3_O_4_)_etc_ showed the same *λ*_max_ value (205 nm).

Band gap values, indicative of the energy required for electronic transitions within the materials, decreased from 5.4 eV in (Co_3_O_4_)_aq_ to 5.3 and 5.1 eV in its nanocomposites rGO@(Co_3_O_4_)_aq_ and rGO@(Co_3_O_4_)_aqc_, respectively. However, there was a significant rise in the band gap of (Co_3_O_4_)_et_ (5.3 eV) to 5.9 and 5.8 eV in its nanocomposites rGO@(Co_3_O_4_)_et_ and rGO@(Co_3_O_4_)_etc_, respectively. The variations in band gaps may be owing to the different nature of plant coatings (aqueous and ethanolic extracts) on the surfaces of synthesized nanomaterials. The smaller and higher bandgap materials have their own advantages and disadvantages. These values provide insights into the material's optical properties and electronic structures, which are crucial for various applications. In water splitting applications, materials with smaller band gap energies are generally more effective.^44^

### 3.5 Scanning electron microscopy (SEM)

**Fig. 7:**
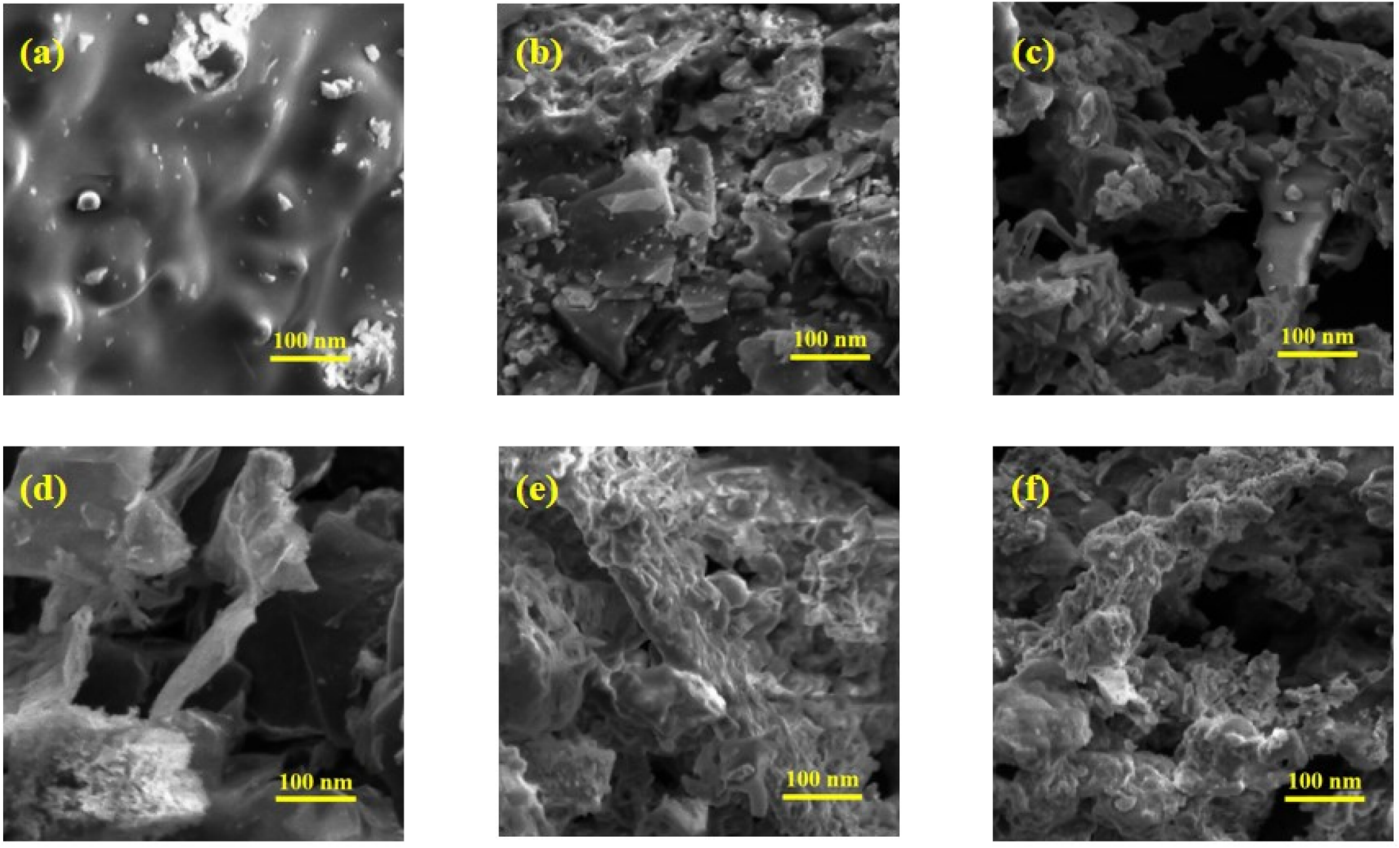
SEM images of (a) (Co_3_O_4_)_aq_, (b) (Co_3_O_4_)_et_, (c) rGO@(Co_3_O_4_)_aq_, (d) rGO@(Co_3_O_4_)_et,_ (e) rGO@(Co_3_O_4_)_aqc_, and (f) rGO@(Co_3_O_4_)_etc_.

SEM analysis was employed to examine the surface morphology of Co_3_O_4_ NPs and their nanocomposites with rGO (Fig. 7a–f). The SEM images indicated that the constituent particles of (Co_3_O_4_)_aq_ and (Co_3_O_4_)_et_ are irregularly shaped spheres and agglomerated together (Fig. 7a and b). The aggregation of particles is influenced by secondary metabolites and chemical components in *P. guajava* leaf extract since individual particles are enveloped and stabilized by bioactive compounds.^45^ Plant extract components appear to cluster due to hydrogen bonding within molecules surrounded by these agents.^45^ To overcome agglomeration, we combined Co_3_O_4_ NPs with reduced graphene oxide (rGO). Doping of Co_3_O_4_ with rGO drastically alters the shapes to the rough and porous surfaces (Fig. 7c and d), thus increasing the electrochemical potential of rGO@(Co_3_O_4_)_aq_ and rGO@(Co_3_O_4_)_et_ compared with their (Co_3_O_4_)_aq_ and (Co_3_O_4_)_et_ counterparts, respectively.^46^ Subsequent calcination of rGO@(Co_3_O_4_)_aq_ and rGO@(Co_3_O_4_)_et_ at 700 °C has led to structural diffusion with reduction in rough morphologies, increase in agglomeration of particles^47^ and consequently, the decrease of electrocatalytic water splitting potential of the resulting rGO@(Co_3_O_4_)_aqc_ and rGO@(Co_3_O_4_)_etc_ nanocomposites (Fig. 7e and f). The results of our SEM analyses were consistent with the catalytic activity assessments.

### 3.6 TGA-DSC analysis

**Fig. 8:**
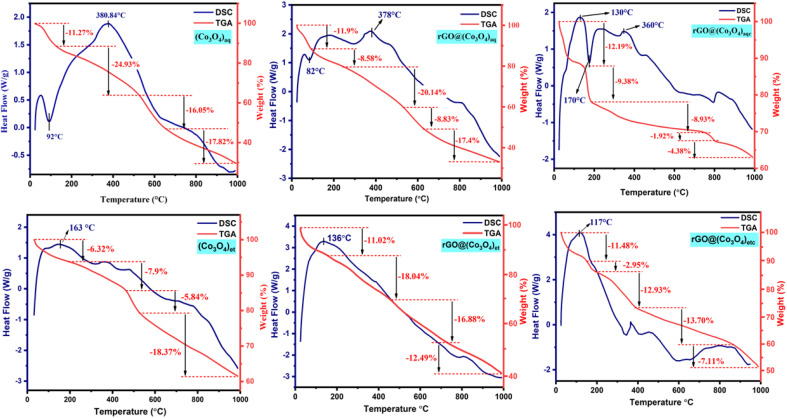
TGA and DTA curve of (Co_3_O_4_)_aq_, rGO@(Co_3_O_4_)_aq_, rGO@(Co_3_O_4_)_aqc_, (Co_3_O4)_et_, rGO@(Co_3_O_4_)_et_, and rGO@(Co_3_O_4_)_etc_.

A Discovery 650 SDT simultaneous thermal analyzer was employed to record thermal data, including TGA and DSC. Thermogravimetric measurements were performed up to a temperature of 1000 °C, with a heating rate maintained at 10 °C min^−1^, whereas an N_2_ (99.999%) atmosphere was maintained throughout the analysis with a flow rate of 50 mL min^−1^ to preclude any potential interference arising from atmospheric air at elevated temperatures. The obtained TGA and DSC curves are shown in Fig. 8.

The obtained results (Fig. 8) reflect the thermal instabilities of the synthesized nanomaterials. According to literature, the initial weight loss at 25–150 °C in the TGA curve, concomitant with an endothermic peak in the DSC curve, is attributed to the evaporation of residual moisture from the investigated samples.^48^ The DSC curves of (Co_3_O_4_)_aq_, rGO@(Co_3_O_4_)_aq_ and rGO@(Co_3_O_4_)_aqc_ show endothermic peaks at 92, 82 and 170 °C, respectively, corresponding to the evaporation of adsorbed water from the surfaces of these nanostructures.^49^ TGA curves also depict the gradual loss of mass corresponding to the exothermic peaks in DSC curves at 380, 378, 130, 163, 136 and 117 °C in (Co_3_O_4_)_aq_, rGO@(Co_3_O_4_)_aq_, rGO@(Co_3_O_4_)_aqc_, (Co_3_O_4_)_et_, rGO@(Co_3_O_4_)_et,_ and rGO@(Co_3_O_4_)_etc_, respectively; these peaks demonstrate the exothermic evolution of residual organic moieties that are present as plant coatings on the surfaces of synthesized nanoparticles. An additional exothermic peak at 360 °C was also displayed in the DSC curve of rGO@(Co_3_O_4_)_aqc_. Conclusively, TGA/DSC analysis shows endothermic loss of moisture and organic contents from the NMs.

### 3.7 Electrocatalytic water splitting applications

#### 3.7.1 OER performance

**Fig. 9:**
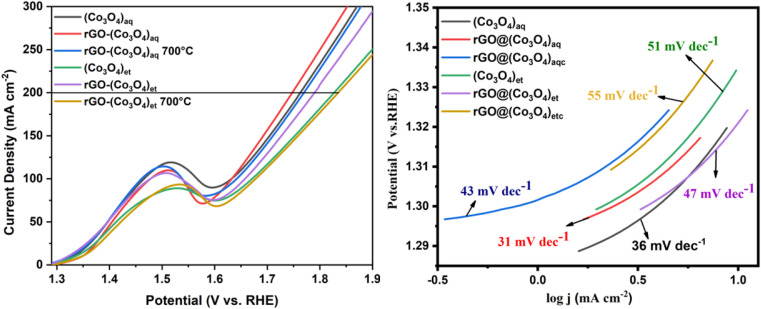
LSV OER curves (left) and corresponding Tafel slopes (right) of (Co_3_O_4_)_aq_, rGO@(Co_3_O_4_)_aq_, rGO@(Co_3_O_4_)_aqc_, (Co_3_O_4_)_et_, rGO@(Co_3_O_4_)_et_, and rGO@(Co_3_O_4_)_etc_.

The LSV OER curves and associated Tafel slopes are shown in Fig. 9. During the oxygen evolution reaction (OER), the aqueous extract-based nanomaterials *i.e.*, (Co_3_O_4_)_aq_, rGO@(Co_3_O_4_)_aq_, and rGO@(Co_3_O_4_)_aqc_ achieved a current density of 200 mA cm^−2^ at a potential of 539, 524, and 546 mV, respectively. The ethanolic aqueous extract based nanomaterials *i.e.*, (Co_3_O_4_)_et_, rGO@(Co_3_O_4_)_et_, and rGO@(Co_3_O_4_)_etc_ required a potential of 604, 566 and 611 mV, respectively to reach a current density of 200 mA cm^−2^ (Fig. 9).

Overall, the OER electrocatalytic potential of the biosynthesized nanomaterials was reduced in the following order:rGO@(Co_3_O_4_)_aq_ > (Co_3_O_4_)_aq_ > rGO@(Co_3_O_4_)_aqc_ > rGO@(Co_3_O_4_)_et_ > (Co_3_O_4_)_et_ > rGO@(Co_3_O_4_)_etc_

The obtained results clarify that all the aqueous extract-based nanomaterials show better OER catalytic performance compared with their ethanolic extract based counterparts; this is mainly because of the differences in the nature of plant coatings (aqueous or ethanolic extract based) on the surfaces of investigated nanomaterials. Moreover, rGO decorated non-calcined rGO@(Co_3_O_4_)_aq_ and rGO@(Co_3_O_4_)_et_ nanocomposites showed superior electrocatalytic water splitting performance compared with their calcined rGO@(Co_3_O_4_)_aqc_ and rGO@(Co_3_O_4_)_etc_ counterparts, respectively. This is due to more rough and porous morphologies of the non-calcined materials compared with their calcined counterparts as discussed in Section 3.5. Calcination decreases the roughness and porosity with a consequent lowering of the electrocatalytic water splitting performance. The obtained results also clarify that the HER electrocatalytic performance of (Co_3_O_4_)_aq_ and (Co_3_O_4_)_et_ is significantly improved after forming their nanocomposites with GO *i.e.*, rGO@(Co_3_O_4_)_aq_ and rGO@(Co_3_O_4_)_et_, respectively but this performance is significantly lowered after calcination at 700 °C in rGO@(Co_3_O_4_)_aqc_ and rGO@(Co_3_O_4_)_etc_.

The Tafel slope is a crucial parameter in electrochemistry, particularly when evaluating the performance of catalysts for OER. It essentially describes the relationship between the overpotential and the current density in an electrochemical reaction, such as OER. The Tafel slope indicates how sensitive the current density is to changes in overpotential. Generally, a smaller Tafel slope indicates a more efficient catalyst, as it means less energy is required to drive a significant increase in current density. The electrocatalytic kinetics of the biosynthesized samples were compared on the basis of their Tafel slopes. The rGO@(Co_3_O_4_)_aq_ nanocomposite displayed the lowest Tafel slope (31 mV dec^−1^) compared with the remaining aqueous extract-based materials *i.e.*, (Co_3_O_4_)_aq_ (36 mV dec^−1^) and rGO@(Co_3_O_4_)_aqc_ (43 mV dec^−1^), verifying that rGO@(Co_3_O_4_)_aq_ demonstrates the most efficient OER kinetics. Among the ethanolic extract-based nanocomposites, the highest OER kinetics was displayed by rGO@(Co_3_O_4_)_et_ (Tafel slope = 47 mV dec^−1^) compared with (Co_3_O_4_)_et_ (51 mV dec^−1^) and rGO@(Co_3_O_4_)_etc_ (55 mV dec^−1^) (Fig. 9).

The potential-dependent Tafel slopes for metal oxides can also be predicted from microkinetic models. A theoretical study on RuO_2_ demonstrated that the rate-determining step is O–O bond formation (*O + H_2_O → *OOH + H^+^ + e^−^) which determined the Tafel slope value of ∼39 mV dec^−1^ at a potential lower than ∼1.5 V_RHE_, where the active coordinatively unsaturated Ru sites (*) is filled with *OH.^50^ Scott *et al.*^51^ observed a Tafel slope of approximately 25 mV dec^−1^ at low overpotentials on Ru-based oxides. They proposed that this low Tafel slope could be attributed to the potential dependence of the coverage of surface species participating in the rate-determining step. This implies that the observed Tafel slope is influenced by the equilibrium coverage of intermediates at low potentials, leading to a distinct mechanistic pathway. Krasil'shchikov's path describes one of the most renowned mechanisms with the corresponding Tafel slopes (eqn (1)–(4)).

1 M + OH^−^ ↔ MOH + e^−^, *b* = 120 mV dec^−1^

2 MOH + OH^−^ ↔ MO^−^ + H_2_O, *b* = 60 mV dec^−1^

3 MO^−^ → MO + e^−^, *b* = 45 mV dec^−1^

4 2MO → 2M + O_2_, *b* = 19 mV dec^−1^

The rate determining step can be determined based on the Tafel slope. NiCo_2_O_4_ and IrO_2_ have Tafel slope values of 59 and 48 mV dec^−1^, respectively corresponding to their rate determining steps as displayed in steps 2 and 3, respectively. The smaller Tafel slope of IrO_2_ demonstrates reduced kinetic overpotential losses and can be owing to the rise of the bond strength for OH^−^ adsorption on IrO_2_, which enhances the rate of the first electron reaction step (eqn (1)), consequently increasing the electrocatalytic kinetics. Moreover, the concentration of active sites and their contribution can be found from the changes in Tafel slopes. The reduced Tafel slope of IrO_2_ may also be owing to the rise of active sites and their active contribution.^52^ In our current study, the observed Tafel slope (31 mV dec^−1^) of rGO@(Co_3_O_4_)_aq_ nanocomposite could indicate a mechanism involving specific adsorption processes, such as OH^−^ adsorption, influencing the rate-determining step. This highlights the importance of considering surface coverage and adsorption phenomena when interpreting Tafel slopes in OER kinetics.^53^

#### 3.7.2 HER performance

**Fig. 10:**
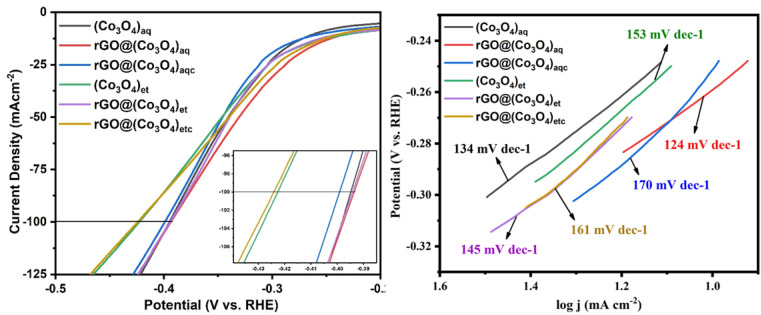
LSV HER curves and corresponding Tafel slopes of (Co_3_O_4_)_aq_, rGO@(Co_3_O_4_)_aq_, rGO@(Co_3_O_4_)_aqc_, (Co_3_O4)_et_, rGO@(Co_3_O_4_)_et_, and rGO@(Co_3_O_4_)_etc_.

The LSV HER electrocatalytic performance decreased in the order of rGO@(Co_3_O_4_)_aq_ > (Co_3_O_4_)_aq_ > rGO@(Co_3_O_4_)_aqc_ among the aqueous extract-derived NMs and rGO@(Co_3_O_4_)_et_ > (Co_3_O_4_)_et_ > rGO@(Co_3_O_4_)_etc_ among the ethanolic extract-derived NMs. The trend of decreasing LSV HER electrocatalytic potential (Fig. 10) was similar to that of the LSV OER pattern (discussed under Section 3.7.1). Thus, the LSV HER and OER results are in good agreement with each other.

The LSV HER experiments show that rGO@(Co_3_O_4_)_aq_, (Co_3_O_4_)_aq_ and rGO@(Co_3_O_4_)_aqc_ require a potential of 704, 708 and 714 mV, respectively to acquire a current density of 100 mA cm^−2^ and the corresponding Tafel slopes were 124, 134 and 170 mV dec^−1^, respectively. On the other hand, rGO@(Co_3_O_4_)_et_, (Co_3_O_4_)_et_ and rGO@(Co_3_O_4_)_etc_ achieved the same current density (100 mA cm^−2^) at the required potentials of 632, 635 and 700 mV, respectively with the corresponding Tafel slopes of 145, 153 and 161 mV dec^−1^, respectively.

A comparison of the Tafel slope values demonstrated that, rGO@(Co_3_O_4_)_aq_ exhibited the lowest Tafel slope value of 124 mV dec^−1^ among the aqueous extract-derived materials, indicating that is has the most efficient HER kinetics compared with (Co_3_O_4_)_aq_ (134 mV dec^−1^) and rGO@(Co_3_O_4_)_aqc_ (170 mV dec^−1^). From the ethanolic extract-based nanomaterials, rGO@(Co_3_O_4_)_et_ demonstrated the most favorable HER performance, requiring 632 mV to reach 100 mA cm^−2^, with the lowest Tafel slope of 145 mV dec^−1^.

Similar to the LSV OER results, the non-calcinated rGO@(Co_3_O_4_)_aq_ and rGO@(Co_3_O_4_)_et_ products show higher HER catalytic performance compared with their calcinated rGO@(Co_3_O_4_)_aqc_ and rGO@(Co_3_O_4_)_etc_ counterparts, respectively. rGO@(Co_3_O_4_)_aq_ and rGO@(Co_3_O_4_)_et_ show higher catalytic potential among their respective series of aqueous and ethanolic extract-derived products, respectively. However, a combined electro-catalytical comparison between all the products clarifies that aqueous extract-derived rGO@(Co_3_O_4_)_aq_ and (Co_3_O_4_)_aq_ products (Tafel slopes = 124 and 134 mV dec^−1^, respectively) are superior HER catalysts compared with their ethanolic extract-derived rGO@(Co_3_O_4_)_et_ and (Co_3_O_4_)_et_ counterparts, respectively (Tafel slopes = 145 and 153 mV dec^−1^, respectively). On the other hand, the ethanolic extract-derived calcined rGO@(Co_3_O_4_)_etc_ product (Tafel slope = 161 mV dec^−1^) has a higher HER performance compared with its aqueous extract-derived calcined rGO@(Co_3_O_4_)_aqc_ counterpart (Tafel slope = 170 mV dec^−1^). Overall, our study recommends that rGO@(Co_3_O_4_)_aq_ and (Co_3_O_4_)_aq_ are more useful HER electrocatalysts and can be applied more successfully for water splitting compared with the remaining nanomaterials.

#### 3.7.3 Double-layer capacitance (*C*_dl_)

**Fig. 11:**
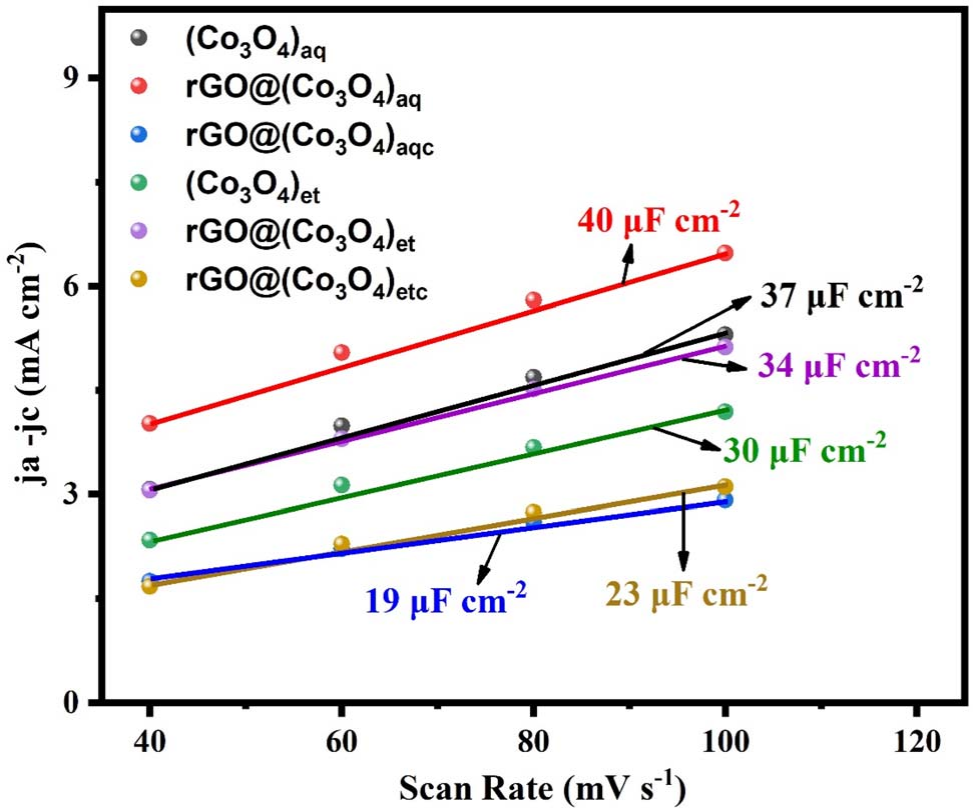
Double layer capacitance (*C*_dl_) values of (Co_3_O_4_)_aq_, rGO@(Co_3_O_4_)_aq_, rGO@(Co_3_O_4_)_aqc_, (Co_3_O4)_et_, rGO@(Co_3_O_4_)_et_, and rGO@(Co_3_O_4_)_etc_.

The cyclic voltammetry (CV) voltammograms (Fig. S3 in ESI†) illustrate clear oxidation–reduction peaks in the synthesized nanoproducts, indicating their reversible redox behavior and potential utility in water splitting. Fig. 11 shows a visual comparison of the *C*_dl_ values of the synthesized samples, which were determined using cyclic voltammograms at various scan rates of 40, 60, 80, and 100 mV s^−1^. In cyclic voltammetry (CV), the double-layer capacitance (*C*_dl_) quantifies the capacitance associated with the electrical double layer formed between the electrode and electrolyte solution interface.^54^ If a sample (electrode) displays a greater *C*_dl_ value in cyclic voltammetry, it typically indicates a larger surface area or a higher concentration of charge carriers in the double layer. The *C*_dl_ values were 40, 37 and 19 μF cm^−2^ for rGO@(Co_3_O_4_)_aq_, (Co_3_O_4_)_aq_ and rGO@(Co_3_O_4_)_aqc_, respectively and 34, 30 and 23 μF cm^−2^ for rGO@(Co_3_O_4_)_et_, (Co_3_O_4_)_et_, and rGO@(Co_3_O_4_)_etc_, respectively. Overall, the *C*_dl_ values decreased in the order of rGO@(Co_3_O_4_)_aq_ > (Co_3_O_4_)_aq_ > rGO@(Co_3_O_4_)_aqc_ among the aqueous extract-derived NMs and rGO@(Co_3_O_4_)_et_ > (Co_3_O_4_)_et_ > rGO@(Co_3_O_4_)_etc_ among the ethanolic extract-derived NMs. Additionally, the findings of our study clarified that the surface area of the non-calcined rGO@(Co_3_O_4_)_aq_ and rGO@(Co_3_O_4_)_et_ nanocomposites is larger compared with that of their calcined rGO@(Co_3_O_4_)_aqc_ and rGO@(Co_3_O_4_)_etc_ counterparts, respectively. Thus, the *C*_dl_ results are in complete agreement with the LSV OER and HER data and reflect the electrocatalytic water splitting potential of the synthesized products. However, rGO@(Co_3_O_4_)_aq_ and rGO@(Co_3_O_4_)_et_ nanocomposites are more suitable electrocatalytic candidates among their respective series of aqueous and ethanolic extract-derived products due to their greater surface areas.

## 4 Conclusion

The aqueous and ethanolic extracts of *Psidium guajava* leaves were successfully employed as reducing, capping and stabilizing agents for the synthesis of cobalt oxide (Co_3_O_4_) nanoparticles and rGO decorated Co_3_O_4_ nanocomposites. FTIR analysis revealed the presence of organic coatings on the surfaces of all biosynthesized NPs. The NMs have crystallite sizes between 10 to 15.4 nm and band gaps within 5.1 to 5.9 mV. TGA/DSC analysis show the endothermic loss of moisture and exothermic removal of organic moieties. SEM images displayed the rough and porous morphologies of rGO decorated Co_3_O_4_ nanocomposites, making them efficient catalysts for water splitting. CV voltammograms reflect clear oxidation–reduction peaks for NMs, demonstrating their reversible redox behavior and potential utility in water splitting. LSV OER and LSV HER results suggested that the incorporation of rGO into Co_3_O_4_ enhances the electrocatalytic water-splitting efficiency, which was decreased after calcination at 700 °C. Moreover, the aqueous extract-derived NMs were electrocatalytically more active compared with their respective ethanolic extract-derived counterparts. All the synthesized nanomaterials were effective for electrocatalytic generation of hydrogen. However, the highest HER/OER electrocatalytic water splitting efficiency was displayed by rGO@(Co_3_O_4_)_aq_. It is recommended that this study is extended to the *Psidium guajava* mediated synthesis of other hybrid nanocomposites of Co_3_O_4_ and can also be applied to the photoelectrocatalytic splitting of water.

## Data availability

All the data will be provided and made available upon request.

## Author contributions

Sumera Akram (investigation, methodology and writing the original draft); Shabbir Hussain (conceptualization, project administration, resources and supervision); Muhammad Arif (conceptualization, resources and co-supervision); Mirza Haider Ali (data curation and writing-review and editing); Muhammad Tariq (formal analysis, software and validation); Abdur Rauf (formal analysis, visualization and validation); Khurram Shahzad Munawar (formal analysis and validation); Hamad M. Alkahtani (funding acquisition and writing-review and editing); Amer Alhaj Zen (visualization and validation); Syed Adnan Ali Shah (formal analysis and writing-review and editing).

## Conflicts of interest

It is hereby declared that there is no conflict of interest among the authors.

## Abbreviations

NPs: Nanoparticles
NCs: Nanocomposites
NMs: Nanomaterials
rGO: Reduced graphene oxide
(Co_3_O_4_)_aq_ and (Co_3_O_4_)_et_: Represent Co_3_O_4_ nanoparticles synthesized with aqueous and ethanolic extracts, respectively, of *P. guajava* leaves
rGO@(CO_3_O_4_)_aq_ and rGO@(Co_3_O_4_)_et_: Indicate the nanocomposites of (Co_3_O_4_)_aq_ and (Co_3_O_4_)_et_, respectively, with reduced graphene oxide (rGO)
rGO@(CO_3_O_4_)_aqc_ and rGO@(Co_3_O_4_)_etc_: Represent the calcined products (at 700 °C) of rGO@(CO_3_O_4_)_aq_ and rGO@(Co_3_O_4_)_et_, respectively

## Supplementary Material

RA-015-D5RA00040H-s001
