# Supplementary material for: Psidium guajava mediated green synthesized cobalt oxide nanoparticles dispersed on reduced graphene oxide for electrocatalytic water splitting
Source: RSC Adv. 2025 May 1;15(18):13786–98. doi: 10.1039/d5ra00040h (PMC12045089; doi:10.1039/d5ra00040h)
Supplement: RA-015-D5RA00040H-s001 [file RA-015-D5RA00040H-s001.pdf]

This journal is © The Royal Society of Chemistry 2025

Supplementary Material

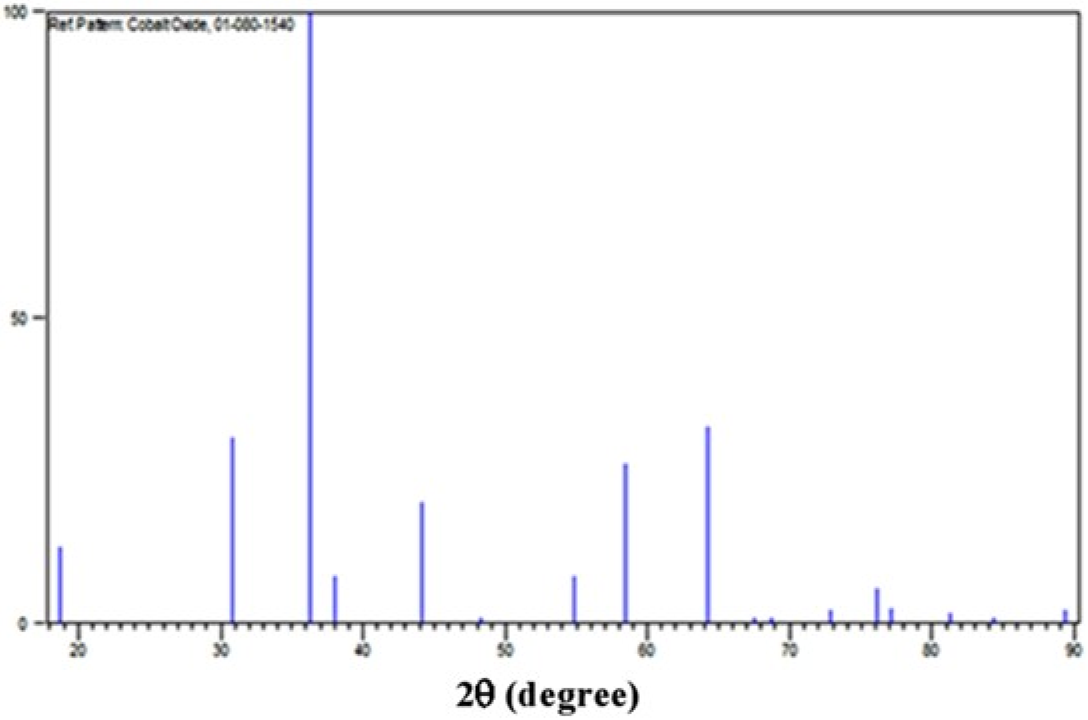

Fig. S1. Reference XRD spectrum of Co_3O_4

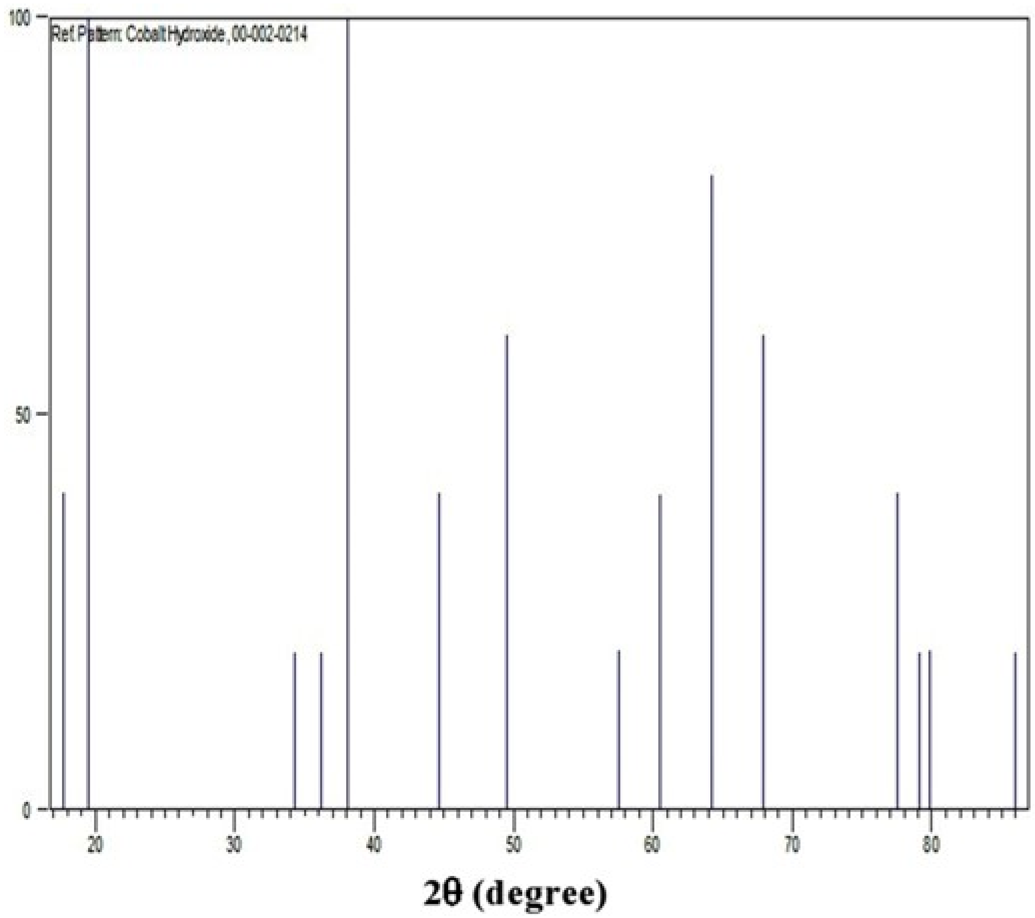

Fig. S2. Reference XRD spectrum of cobalt hydroxide

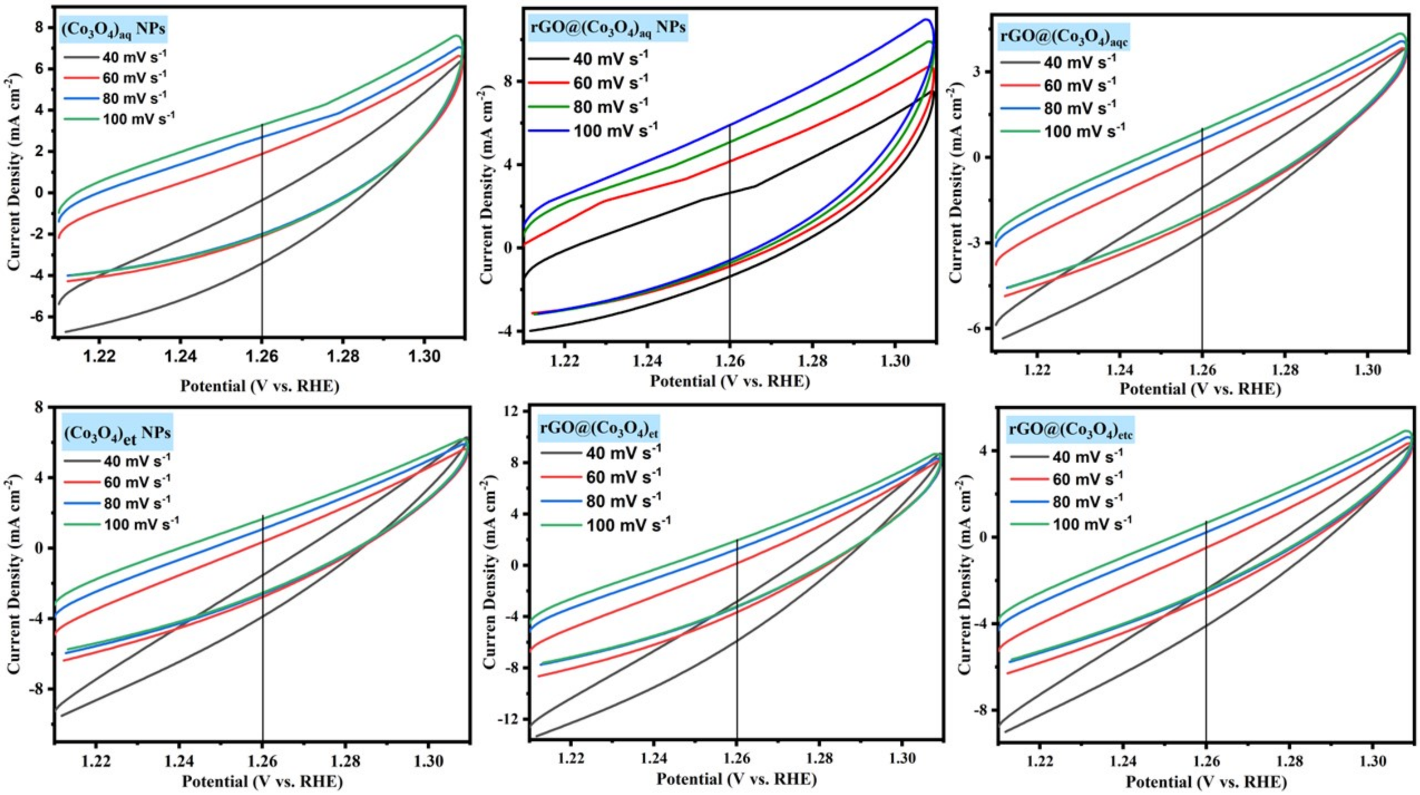

Fig. S3. Cyclic voltammograms of $(Co_3O_4)_{aq}$, rGO@$(Co_3O_4)_{aq}$, rGO@$(Co_3O_4)_{aqc}$, $(Co_3O_4)_{et}$, rGO@$(Co_3O_4)_{et}$ and rGO@$(Co_3O_4)_{etc}$
